# Short-term arginine deprivation results in large-scale modulation of hepatic gene expression in both normal and tumor cells: microarray bioinformatic analysis

**DOI:** 10.1186/1743-7075-3-37

**Published:** 2006-09-08

**Authors:** Hwei Xian Leong, Carl Simkevich, Anne Lesieur-Brooks, Bonnie W Lau, Celine Fugere, Edmond Sabo, Nancy L Thompson

**Affiliations:** 1 Division of Hematology and Oncology, Dept. of Medicine, Rhode Island Hospital – Brown Medical School, Providence, RI, USA; 2 COBRE Center for Genetics and Genomics, Division of Biology and Medicine, Brown University, Providence, RI, USA; 3 Dept. Pathology and Laboratory Medicine, Rhode Island Hospital – Brown Medical School, Providence, RI, USA

## Abstract

**Background:**

We have reported arginine-sensitive regulation of LAT1 amino acid transporter (SLC 7A5) in normal rodent hepatic cells with loss of arginine sensitivity and high level constitutive expression in tumor cells. We hypothesized that liver cell gene expression is highly sensitive to alterations in the amino acid microenvironment and that tumor cells may differ substantially in gene sets sensitive to amino acid availability. To assess the potential number and classes of hepatic genes sensitive to arginine availability at the RNA level and compare these between normal and tumor cells, we used an Affymetrix microarray approach, a paired *in vitro *model of normal rat hepatic cells and a tumorigenic derivative with triplicate independent replicates. Cells were exposed to arginine-deficient or control conditions for 18 hours in medium formulated to maintain differentiated function.

**Results:**

Initial two-way analysis with a p-value of 0.05 identified 1419 genes in normal cells versus 2175 in tumor cells whose expression was altered in arginine-deficient conditions relative to controls, representing 9–14% of the rat genome. More stringent bioinformatic analysis with 9-way comparisons and a minimum of 2-fold variation narrowed this set to 56 arginine-responsive genes in normal liver cells and 162 in tumor cells. Approximately half the arginine-responsive genes in normal cells overlap with those in tumor cells. Of these, the majority was increased in expression and included multiple growth, survival, and stress-related genes. GADD45, TA1/LAT1, and caspases 11 and 12 were among this group. Previously known amino acid regulated genes were among the pool in both cell types. Available cDNA probes allowed independent validation of microarray data for multiple genes. Among genes downregulated under arginine-deficient conditions were multiple genes involved in cholesterol and fatty acid metabolism. Expression of low-density lipoprotein receptor was decreased in both normal and tumor cells.

**Conclusion:**

Arginine-sensitive regulation appears to be an important homeostatic mechanism to coordinate cell response and nutrient availability in hepatic cells. Genes predicted as arginine-responsive in stringent microarray data analysis were confirmed by Northern blot and RT-PCR. Although the profile of arginine-responsive genes is altered and increased, a considerable portion of the "arginome" is maintained upon neoplastic transformation.

## Background

Cell growth is dependent upon availability of essential amino acids for protein synthesis and this relationship makes amino acid-dependent control of gene expression an important area of study [1-3]. We previously reported that levels of the tumor-associated glycoprotein amino acid transporter, TA1/LAT1/CD98 light chain/SLC7A5, increase in normal hepatic cells under low arginine conditions, while levels are constitutive and high in hepatic tumor cells [4,5]. Upregulation of this gene is associated with multiple cancer types and we and others have hypothesized that increased expression may provide an adaptive advantage in the tumor microenvironment where nutrients are limiting [6,7]. Loss of nutrient-sensitive regulation may comprise a subset of the loss of responsiveness to negative growth signals or autonomy from positive growth factors, characteristic of cancer cells in general [8]. We thus manipulated arginine concentration in a culture medium that maintains hepatic differentiation as a means to investigate, using a paired normal and tumorigenic cell type, how many and what types of hepatic genes are responsive to a transient change in amino acid levels. Affymetrix gene chips and bioinformatic analyses were used in a nutrigenomics approach. The goals of the study were to: 1) assess the scope of arginine-responsive hepatic gene expression using a well-defined *in vitro *rat model of normal and tumorigenic cells, 2) determine to what extent amino acid responsive regulation is retained upon transformation, and 3) provide a microarray dataset predicting novel genes and pathways subject to amino acid (arginine) regulation.

## Results and discussion

Triplicate RAE 230 arrays containing a total of 15,923 rat genes were hybridized for both normal and tumor cells grown under arginine-sufficient (+) and arginine-deficient (-) conditions for 18 hours in three independent pair-wise comparison experiments. Microarray Suite™ 5.0 software analysis of the resulting datasets revealed a mean of 8611 genes or 54% of the total rat array scored "present" (expressed) for normal cells versus 8355 genes or 53% in tumor cells (Table 1). Of the genes expressed, pair-wise comparisons of Arg + and - conditions revealed no change in expression for 7042 (82%) of genes in normal cells versus 6173 (74%) in tumor cells. Using a p-value of 0.05 or less and pair-wise comparisons for the three experiments in normal cells, expression of 811 genes increased, 608 decreased and 97 showed marginal change relative to arginine-sufficient controls. For tumor cells, transient arginine deficiency resulted in increased expression of 1249 genes, decreased expression of 926 genes, and marginal changes in 109. Thus, expression of as many as 2175 genes was scored as arginine-sensitive when the data were analyzed as independent pairs. This represented 8.9% of genes in normal cells and 13.6% of genes in tumor cells. At the time of the data collection, nearly half of these genes lacked complete functional annotation in the rat genomic database.

**Table 1:** Microarray Suite analysis of gene regulation in normal vs. tumor cells in response to 18 hr arginine deprivation.

**CODE**	**CELL TYPE**	**ARG STATUS**	**ABSENT**	**PRESENT**	**NET INCREASE**	**NET DECREASE**	**MARGINAL**	**NO CHANGE**
**N1**	NORMAL	Arg +	7189	8474				
**N3**	"	Arg +	7102	8536				
**N5**	"	Arg +	7010	8662				
								
**N2**	"	Arg deficient	7140	8532	1242	876	135	6221
**N4**	"	Arg deficient	7296	8381	358	136	52	7990
**N6**	"	Arg deficient	6601	9082	832	811	105	6914
								
**MEAN**	**Normal**		**7106**	**8611**	**811**	**608**	**97**	**7042**
AVEDEV			167	174	302	314	30	632
								
**T1**	TUMOR	Arg +	7085	8535				
**T3**	"	Arg +	7051	8607				
**T5**	"	Arg +	7423	8224				
								
**T2**	"	Arg deficient	6934	8725	1514	1187	119	5715
**T4**	"	Arg deficient	7664	7973	1391	913	117	6186
**T6**	"	Arg deficient	7573	8065	843	678	86	6617
								
**MEAN**	**Tumor**		**7288**	**8355**	**1249**	**926**	**109**	**6173**
AVEDEV			265	268	271	174	14	305

Numbers of rat genes in Affymetrix RAE 230 array demonstrating expression, no change, increase, decrease and marginal change. Change in gene expression based on p value of 0.05 or less in pair-wise comparisons of three independent datasets. RAE 230 array contains 15,923 genes.

Because the number of genes in the preliminary analysis comprised a large portion of the rat genome, additional filters were applied to the dataset using GeneSpring™ 6.1 and 7.0 software analysis in order to identify the most reproducible and significant alterations resulting from transient arginine deficiency. For each cell type, all + samples were compared to all – samples, resulting in 9 comparisons. A concordance threshold of 66% or greater was set and 'arginine-responsive' increases or decreases were defined as a change of 2-fold and higher under arginine-deficient conditions. All other genes expressed were defined as "no change".

**Figure 1:**
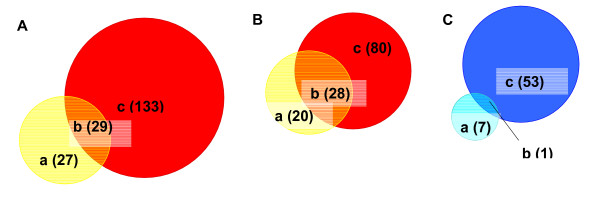
**Comparison of arginine-responsive gene sets in normal and tumor cells**. (A) a: Arginine-responsive genes in Normal Rat Hepatic Cells; b: Arginine-responsive genes in both Normal and Tumor Rat Hepatic Cells; c: Arginine-responsive genes in Tumor Rat Hepatic Cells. (B) a: Genes with expression Increase in Normal b: Genes with expression Increase in both Normal and Tumor; c: Genes with expression Increase in Tumor. (C) a: Genes with expression Decrease in Normal; b: Genes with expression Decrease in both Normal and Tumor; c: Genes with expression Decrease in Tumor.

Using these more stringent parameters, the total number of arginine-responsive genes in normal cells and tumor cells was significantly reduced, to 56 and 162 respectively. A summary of the data comparing normal and tumor cells by functional gene class and direction of change is shown in Table 2. All 56 and 162 genes in normal and tumor cells respectively listed by accession numbers and corresponding direction of change relative to arginine-sufficient conditions (I = increase; D = decrease) are listed in supplemental Tables 4 and 5 [see additional files 1 and 2]. The entire 12 microarray dataset is available in MIAME format [9] in the NCBI Gene Expression Omnibus (GEO) database, confirmation #GSE2275. Figure 1 presents Venn diagrams of overlap between the arginine-responsive gene subsets of normal and tumor cells. Just over half the arginine-responsive genes in normal cells are also responsive in tumor cells. A surprising finding was the large number (133) of additional genes demonstrating arginine-responsive expression in tumor cells. More than one third of these were listed as transcribed sequences with no listed functional information or homology to known sequences at the time of analysis. Not surprisingly, genes with increased expression upon arginine depletion in both normal and tumor cells include a large number of genes involved in cell cycle and growth regulation, cell death and apoptosis, and stress response. Several of these were known amino acid-responsive genes such as asparagine synthetase [10]. Among other genes scoring as increased in both cell types was growth-arrest DNA damage-inducible 45 alpha (GADD45) and caspase 12. Genes down-regulated in both normal and tumor cells include plasminogen activator urokinase and low density lipoprotein receptor. Genes that exhibited arginine-responsive regulation in normal cells but a loss in tumor cells (or vice versa) may represent 'interesting' subsets in which the potential contribution of amino-acid regulation to the malignant phenotype could be explored. Genes whose expression was altered in tumor cells but not normal cells included tumor suppressor retinoblastoma-like 2, stress-response protein 70 kDa heat shock protein precursor and cell-surface linked signal transducer MAP-kinase phosphatase, cell cycle regulator cyclin dependent kinase inhibitor 2C (p18) and Ras. Genes scoring as changed in normal but "no change" in tumor cells were mainly in cholesterol and steroid biosynthesis pathways. Related to these were farnesyl diphosphate synthase and insulin-induced gene 1/growth response protein CL-6 (Insig-1/CL-6), an ER-residing membrane protein that blocks the proteolytic activation of sterol-regulatory element binding protein (SREBPs) transcription factors that activate the synthesis of fatty acids and cholesterol [11-13].

**Table 2:** Stringent GeneSpring analysis of gene regulation in normal vs. tumor cells in response to 18 hr arginine deprivation.

Class (n)	Example	Change in Normal	Change in Tumor
Cell Growth and Maintenance (10)	growth arrest and DNA-damage-inducible 45 alpha Rattus norvegicus transcribed sequence with moderate similarity to protein sp:Q9H3K2 (H.sapiens) DER2_HUMAN Dermal papilla derived protein 2 (AA858928) neuronal cell death inducible putative kinase (NIPK) Caspase 12 tumor-associated protein 1 solute carrier family 7, member 1 solute carrier family 3, member 2 homocysteine-inducible, endoplasmic reticulum stress-inducible ubiquitin-like domain member 1 asparagine synthetase CTL target antigen	**Inc**	**Inc**
Unclassified (1)	Low density lipoprotein receptor	**Dec**	**Dec**
Cell Growth and Maintenance (28)	retinoblastoma-like 2 epithelial calcium channel 1 sequestosome 1 70 kda heat shock protein precursor protein arginine N-methyltransferase 3(hnRNP methyltransferase S. cerevisiae)-like 3 eukaryotic translation initiation factor 4E binding protein 1 protease, serine, 15 B-cell translocation gene 1 5-hydroxytryptamine (serotonin) receptor 3a MAP-kinase phosphatase (cpg21) diphtheria toxin receptor	**NC**	**Inc**
Unclassified (24)	growth arrest specific 5		
Cell Growth and Maintenance (12)	cyclin dependent kinase inhibitor 2C solute carrier family 6, member 6 fatty acid Coenzyme A ligase, long chain 3 Inhibitor of DNA binding 3, dominant negative helix-loop-helix protein Topoisomerase (DNA) 2 alpha adducin 3, gamma	**NC**	**Dec**
Signal Transduction (2)	RAB3D, member RAS oncogene family		
Developmental Processes (1)	Sperm-associated antigen 5 arginine vasopressin receptor 1A GATA binding protein 6		
Unclassified (21)	Nesprin-1		
Cell Growth and Maintenance (1)	endo-alpha-mannosidase	**Inc**	**NC**
Signal Transduction (2)	Inhibitor of DNA binding 2, dominant negative helix-loop-helix protein Rattus norvegicus transcribed sequence with strong similarity to protein pir:I55595 (H. sapiens) I55595 splicing factor (BG372903)		
Unclassified (7)	transmembrane 4 superfamily member 3 (NM_133526)		
Cell Growth and Maintenance (6)	cytochrome P450, subfamily 51 7-dehydrocholesterol reductase isopentenyl-diphosphate delta isomerase sterol-C4-methyl oxidase-like] farensyl diphosphate synthase Growth Response protein CL-6 (Insig-1)	**Dec**	**NC**

'Change' defined as 2 fold and higher increase (+) or decrease (-) in expression from + arg to - arg. 'NC' (No change) defined as all other genes that were *not *differentially expressed with a 2 fold and higher change. Main functional classes are shown in the table, with corresponding representative genes in each class. Categorization of genes was performed using GeneSpring software. The number of genes in each class is also shown beside the class in parentheses.

**Figure 2:**
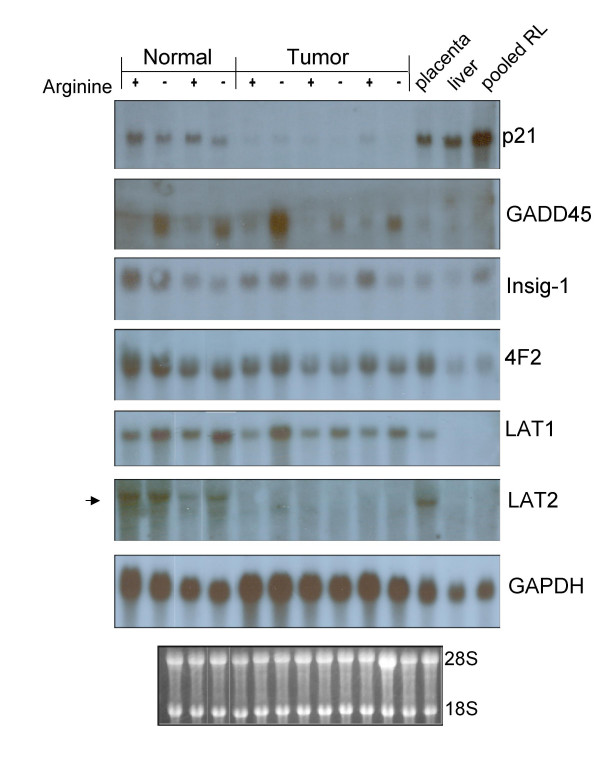
**Northern blot confirmation of selected rat hepatic gene expression in normal and tumor cells in response to 18 hr arginine deprivation**. Total RNA (10 ug per culture condition) was electrophoresed and blotted to nylon membranes for sequential hybridization with P-32 labeled cDNA probes for p21, GADD45, Insig-1, 4F2, LAT1, LAT2 and GAPDH. Autoradiographic exposures shown represent maximal differences observed between + and - arginine conditions. The ethidium bromide stained blot prior to hybridization is shown below for comparison of loading between lanes and evidence of intact RNA quality.

**Figure 3:**
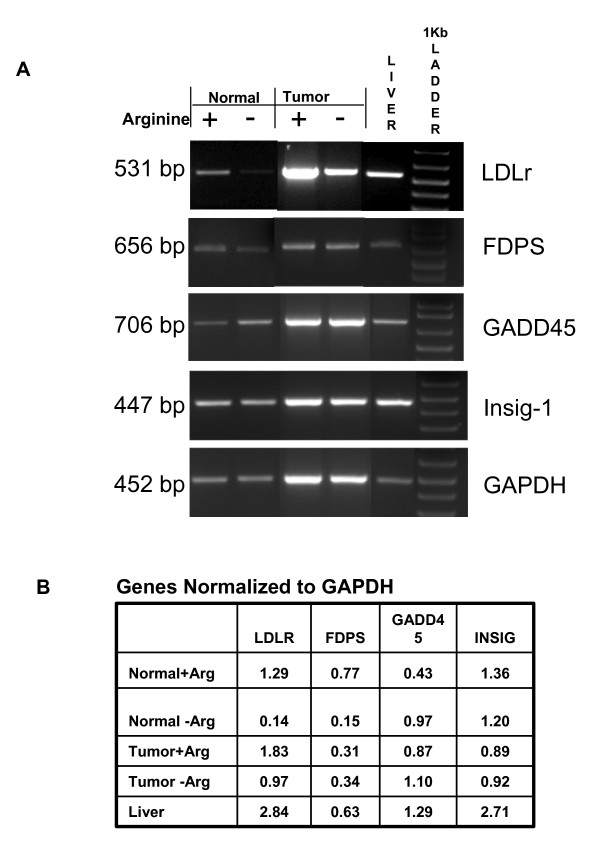
**RT-PCR confirmation of selected rat hepatic gene expression in normal and tumor cells in response to 18 hr arginine deprivation**. Semi-quantitative Reverse Transcription Polymerase Chain Reaction was used to assess relative expression of rat LDLr, farnesyl diphosphate synthase (FDPS), GADD45, Insig-1, and GAPDH for cells cultured with (+) or without (-) arginine, Adult liver served as a positive control. DNase treated total RNA was reverse-transcribed with and without (negative control) reverse transcriptase. **A**. Electrophoresis of PCR products was performed in 2% agarose, 1× TAE gel and visualized by ethidium bromide staining; 1 kb DNA marker was used to verify size of the PCR products. **B**. Densitometry of gel bands was assessed via LabWorks Software and values were ratioed to GAPDH to provide relative comparisons between Arginine + and Arginine - conditions.

Independent verification of expression was sought for a subset of genes via Northern blot analysis (Figure 2) and RT-PCR (Figure 3). Genes were selected in which cDNA probes were available with appropriate known controls to validate selected cases in which arginine-deprivation predicted an increase, decrease or no significant change in expression. Though the level of expression for some genes was found to differ substantially between normal and tumor samples, between arginine + and - conditions respectively, or between independent experimental replicates, trends predicted in microarray data analysis were generally validated by the confirmatory RNA expression analysis and densitometry. That is, LAT1, GADD45 and 4F2 increased in both cell types under conditions of arginine deprivation; LDLr decreased in both cell types upon arginine deprivation; LAT2 and GAPDH showed no significant change in either cell type, though LAT2 expression was very low in tumor cells; p21 decreased in normal cells but was below detection limits in tumor cells; farnesyl diphosphate synthase (FDPS) decreased in normal cells but not in tumor cells upon arginine deprivation; Insig 1 showed large variability with decreases in some replicates but not in others. The use of different probe sets between the microarray and Northern or PCR analyses, differences in quantitation and normalization methodology or undetermined other variables may account for differences in fold change values observed between these techniques.

Given its enormous potential for insight into regulatory physiology, nutrigenomics, the use of genomic tools to study nutrient-gene interactions, is an area of expanding interest in health and disease [14-16]. We found global changes in gene expression when hepatic cells were subjected to short term deficiency of arginine. Arginine, a precursor of proteins and other compounds critical to normal metabolism, is considered essential for growth and development of many mammals [17,18]. Our experimental system was one of amino acid imbalance, but not of total arginine starvation, since the medium was supplemented with 5% non-dialyzed fetal bovine serum and the exposure to deficient conditions was less than 24 hr. In fact, arginine-free medium has been widely used as a means to deplete primary hepatocyte cultures of small numbers of contaminating nonparenchymal cells because all the enzymes necessary for arginine synthesis are expressed by the normal hepatocyte [17]. Importantly, viability was well preserved over the experimental timeframe (data not shown). Thus, the observed changes in gene expression were not the result of impending cell death but are more likely a response to nutrient stress.

Stress caused by changes in the cellular environment including nutrients is recognized as an important physiological parameter to which eukaryotic cells have evolved complex cellular responses [19-22]. Although it is not possible to differentiate primary effects from secondary during this timeframe, overall, cell growth and maintenance, metabolism, stress-related and apoptotic gene classes appear most predominant. Genes associated with metabolism and stress response that were present in the rat chip and showed a significant p-value for altered expression upon arginine deprivation are presented in Table 3. Because many genes associated with stress response in human cells were not present or identifiable on the RAE 230 chip however, it is not possible to fully assess arginine response of these gene sets in this model. With the exception of a subset of genes involved in cholesterol and steroid biosynthesis, there was no overall loss of amino-acid responsive regulation in the tumor cells compared to normal cells. In fact, to our surprise, in assessing the entire rat transcriptome a larger proportion of genes was found to be arginine-responsive in tumor cells compared to the normal cell from which they were derived. The use of this paired cell set in the experimental design is an important consideration as it minimizes variation due to other factors such as genetic strain or viral exposure. Our data not only add to the nutrigenomics literature and database in general, but also extend the finding of others in which changes in amino acid composition or starvation have been associated with specific patterns of hepatic gene response both *in vivo *and *in vitro *[23-25]. The data suggest that arginine deprivation of hepatic normal and tumor cells may induce an initial stress response very similar to the well known endoplasmic reticulum (ER) stress-response described in eukaryotic cell types [19]. The location of products of several arginine-responsive genes including caspase 12, LDLr, Insig-1 and cholesterol/steroid biosynthesis all in the ER are consistent with the induction of a potent luminal ER stress response in both normal and tumor cells. Interestingly, although both cell types appear able to modulate LDLr, a regulator of cholesterol and steroid biosynthesis, in response to arginine deprivation, at some point downstream of the stress pathway, tumor cells appear to deviate by losing regulation of Insig-1 and metabolic genes directly involved in cholesterol and steroid biosynthesis. While it is tempting to speculate that loss of regulation within this gene cluster may be adaptive in a tumor microenvironment of limited nutrient availability, permitting malignant cells to persist in membrane synthesis required for repeated cell divisions, endocytosis, fusion or other processes, the relevance of these observed changes in gene expression *in vitro *to potential alterations in hepatic function and tumor growth *in vivo *are currently unknown. This hypothesis is testable but beyond the limits of the current project. The generation and reporting of nutrigenomic data is undeniably descriptive. However, datasets such as this one are valuable in that they facilitate hypothesis generation and further research. Much more study is needed to define specific genes and mechanistic pathways linking amino acids and other dietary nutrients to cancer risk.

**Table 3:** Arginine sensitivity of rat hepatic cells for known stress response-linked genes.

**A. Normal Hepatic Cells**				
Annotation	Gene name	Affymetrix ID	Change	P value

Metabolism (Cholesterol biosynthesis)	farensyl diphosphate synthase	1370808_at	↓	0.00002
	farnesyl diphosphate farnesyl transferase 1	1387119_at	↓	0.00011
	3-hydroxy-3-methylglutaryl-Coenzyme A synthase 1	1373243_at	↓	0.00002
	cytochrome P450, subfamily 51	1368232_at	↓	0.00002
	mevalonate pyrophosphate decarboxylase	1375852_at	↓	0.00043
	7-dehydrocholesterol reductase	1386990_at	↓	0.00013
	mevalonate kinase	1388218_at	↓	0.00002
	isopentenyl-diphosphate delta isomerase	1367839_at	↓	0.00002
	diaphorase 1	1372012_at	↓	0.00077
	Sterol 14 alpha-demethylase (CYP51)	1387020_at	↓	0.00002
	3-hydroxy-3-methylglutaryl-Coenzyme A reductase	1367932_at	↓	0.00002
	phenylalkylamine Ca2+ antagonist (emopamil) binding protein	1367667_at	↓	0.00021
	cytochrome P450, subfamily 51	1368189_at	↓	0.00002
	mevalonate kinase	1368878_at	↓	0.00120
	low density lipoprotein receptor	1388872_at	↓	0.00021
	isopentenyl-diphosphate delta isomerase	1368020_at	↓	0.00002
Metabolism and energy pathways	ATP citrate lyase	1367854_at	↓	0.00002
Metabolism (Urea Cycle)	arginase 1	1368266_at	↓	0.000492
	glutamate dehydrogenase 1	1370200_at	↓	0.00002
	heterogeneous nuclear ribonucleoprotein A/B	1367754_s_at	↓	0.001651
	ornithine aminotransferase	1367729_at	↓	0.002753
	argininosuccinate lyase	1368916_at	↓	0.015426
Growth arrest and DNA damage inducible gene	Gadd45g-predicted	1388792_at	↓	0.00024
	Gadd45b-predicted	1372016_at	↓	0.00778
	Gadd45gip_1predicted	1371896_at	↓	0.00077
Stress Response	growth response protein (CL-6)	1367894_at	↓	0.00002
	voltage-dependent anion channel 1	1386909_a_at	↓	0.00107
Apoptosis	ankyrin-like repeat protein	1367664_at	↓	0.00005

**B. Hepatic Tumor Cells**				

Annotation	Gene name	Affymetrix ID	Change	P value

Metabolism (Cholesterol biosynthesis)	farnesyl diphosphate farnesyl transferase 1	1367839_at	↓	0.00110
	3-hydroxy-3-methylglutaryl-Coenzyme A synthase 1	1367932_at	↓	0.00002
	cytochrome P450, subfamily 51	1367979_s_at	↓	0.00014
	7-dehydrocholesterol reductase	1368189_at	↓	0.00043
	isopentenyl-diphosphate delta isomerase	1368878_at	↓	0.00004
	protein kinase, AMP-activated, alpha 1 catalytic subunit	1369104_at	↓	0.05055
	diaphorase 1	1370808_at	↓	0.00304
	Dhcr24-predicted	1372012_at	↓	0.01202
	phenylalkylamine Ca2+ antagonist (emopamil) binding protein	1386990_at	↓	0.00165
	cytochrome P450, subfamily 51	1387020_at	↓	0.00650
	low density lipoprotein receptor	1388218_at	↓	0.00002
	mevalonate pyrophosphate decarboxylase	1368020_at	↑	0.03578
Metabolism and energy pathways	ATP citrate lyase	1367854_at	↓	0.00002
Metabolism (Urea Cycle)	glutamate dehydrogenase 1	1370200_at	↓	0.00359
	glutamate dehydrogenase 1	1387878_at	↓	0.00021
	ornithine aminotransferase	1367729_at	↓	0.00002
	arginase 1	1368266_at	↓	0.00061
Growth arrest & DNA damage inducible gene	Gadd45g-predicted	1388792_at	↓	0.00249
	Gadd45gip_1predicted	1371896_at	↓	0.00133
Stress Response	oxygen regulated protein (150 kD)	1370665_at	↓	0.00141
	solute carrier family 2, (facilitated glucose transporter) member 8	1368286_at	↓	0.00049
	growth response protein (CL-6)	1367894_at	↓	0.00004
	insulin induced gene 2	1389377_at	↑	0.02475
	protease, serine, 25	1367478_at	↑	0.00004
Apoptosis	ankyrin-like repeat protein	1367664_at	↓	0.01202
	glycogen synthase kinase 3 beta	1370267_at	↓	0.00249
	valosin-containing protein	1367455_at	↓	0.03841

Genes were selected based on key word categories/annotations shown and search of Affymetrix rat gene chip probes. Not all genes associated with stress response in human cells are included or identifiable in the rat gene chip. Genes scoring as absent in all 6 normal or tumor samples were eliminated from further analysis. For genes scoring as present, the average value of (+) arginine and (-) arginine was calculated for three replicate samples. Direction of change in expression upon arginine deprivation and the associated p value for such change is presented.

## Methods

### Cell culture

The rat hepatocyte normal diploid cell line WB344 ("normal") and GP7TB, a chemically-transformed derivative capable of generating hepatic tumors upon transplantation to syngeneic rats ("tumor"), were obtained from the laboratory of Dr. William Coleman, Dept. Pathology, University of North Carolina, Chapel Hill and maintained as previously described [5]. To assess arginine-responsive gene expression, at no more than 80% confluence, media was removed and cells were rinsed briefly with a custom formulation of Chee's Essential Medium (CEM) without arginine (GIBCO Invitrogen Corp., Carlsbad, CA). Medium was then replaced with CEM with or without L-Arginine HCl at 0.168 gm/liter for an additional 18 hours at 37 C and 5% CO_2_. Media included 5% fetal bovine serum. The CEM media formulation was utilized because it has been demonstrated to maintain differentiated function and gene expression in cultured hepatic cells [4].

### RNA isolation and microarray analysis

Cells were harvested by lysis-extraction of total RNA with TriReagent (Molecular Research Center, Inc., Cincinnati, OH). RNA quantity and quality were assessed by absorbance at 260 and 280 nm using a Genesys 5 spectrophotometer (Spectronic, Leeds, UK). Electrophoresis of the RNA samples followed by ethidium bromide staining was used to further evaluate RNA samples. Only non-degraded RNAs without DNA contamination were utilized. Five μg of total RNA were converted to the first strand of cDNA by using SuperScript II RT (Invitrogen Corporation, Carlsbad, CA), and gel-purified T7-oligo dT(24) (W.M. Keck Foundation, New Haven, CT) as the primer. Second strand synthesis was performed using E coli Polymerase I, DNA ligase, E coli RNA H and T4 DNA Polymerase according to the manufacturer's instructions and reagents in the SuperScript Double-Stranded cDNA Synthesis Kit (Invitrogen). The resulting cDNA was purified by extraction with (25:24:1) phenol: chloroform: isoamyl alcohol (Ambion, Inc., Austin, TX), separation of the aqueous phase using phase lock gels and precipitation in ethanol. The purified cDNA subsequently served as template for production of biotin labeled cRNA transcript using the BioArray High Yield RNA Transcript Labeling Kit (ENZO Biochemical, New York, NY) and biotin-labeled UTP and CTP. Labeled cRNA was isolated using the RNeasy Mini Kit columns (Qiagen, Valencia, CA), quantified for purity, concentration and yield and subsequently fragmented in 100 mM potassium acetate-30 mM potassium acetate-40 mM Tris-acetate (pH 8.1) for 35 minutes at 94 C to generate 35–200 bp fragments, suitable for hybridization. Eukaryotic Target Hybridization was accomplished with 10 μg of fragmented cRNA, eukaryotic hybridization controls, herring sperm DNA (Promega Biosciences, Inc., San Luis Obsipo, CA), acetylated BSA (Invitrogen), 2× hybridization buffer and DEPC treated water to make the cocktail final concentration 100 mM MES and 1 M [Na+]. Following clarification of the cocktail by heating and centrifugation, rat 230A expression arrays (Affymetrix, Santa Clara, CA) were loaded with the target cRNA cocktail and rotated at 60 rpm for 16 hours at 45 C. Following hybridization, the cocktail was removed and the microarrays were washed with Non-stringent buffer [6× SSPE, 0.01% Tween 20 (Pierce, Rockford, IL) 0.005% Antifoam (Sigma, St Louis, MO)] at 30 C and Stringent buffer (100 mM MES, 0.1 M [Na+], 0.01% Tween 20) at 50 C in a Affymetrix GeneChip Fluidics Station 400 according to the manufacturer's protocol. Microarrays were subsequently stained with Streptavidin-conjugated Phycoerythrin (SAPE, Invitrogen, Carlsbad, CA) and staining intensity was antibody amplified using a biotinylated anti-streptavidin antibody (Vector Laboratories, Burlingame, CA) according to Affymetrix protocols. The GeneChips were scanned at 570 nm using the Agilent Technologies G2500A GeneArray scanner with MicroArray Suite software. All hybridization steps were performed at the Brown University COBRE Center for Genomics and Proteomics Microarray Facility. Each hybridization was performed in triplicate from separate RNA prepared from independent cell cultures of both normal and tumor cells.

### Data analysis

Each gene on the 230A array is represented by 20 different 25-base cDNA oligonucleotides complementary to a cRNA target transcript (perfect match) together with specificity control oligonucleotides containing a single base substitution (mismatch) for each perfect-match. The combination of perfect-match and mismatch cDNA oligonucleotides for each gene is termed a probe set. Affymetrix-defined absolute mathematical algorithms describing perfect-match and mismatch hybridization intensities were used to define each gene as "present" or "absent" and assign a value. Binding intensity values were scaled to evaluate differential expression +/- arginine supplementation. Affymetrix-defined comparison mathematical algorithms determined whether a transcript was classified as "increased", "decreased", "marginally increased", "marginally decreased" or "not changed" and a fold change in expression was calculated. Only genes that had signal present were selected from the normalized data for further analysis. This was accomplished using GeneSpring version 7.0 (Silicon Genetics, Redwood City, CA) importing data from Affymetrix MicroArray Suite (MAS 5.0) into GeneSpring as tab delimited text files. Additional criteria/filters were used to classify a gene as significantly up- or down-regulated upon short term arginine starvation: (i) expression of a gene must be classified as increased or marginally increased (or decreased or marginally decreased) in each replicate compared to the arginine sufficient condition. (ii) The mean fold change for arginine starvation-induced gene expression must be greater than 2. Mean transcriptional expression of a given gene was calculated as the sum of the fold change in gene expression for each arginine-minus condition compared to each arginine-sufficient (normal) condition divided by 6. Standard errors in mean transcriptional expression of a gene were also calculated. Comparison between gene expression levels among groups was done using the Two-Way ANOVA test.

All microarray data were submitted in compliance with the Minimal Information about Microarray Experiments (MIAME) via the Gene Expression Omnibus (GEO) data repository under the title "A Bioinformatic Analysis of Arginine-Sensitive Regulation of rat Hepatic Gene Expression", confirmation #GSE2275 [26]. The MIAME standard, developed by the Microarray Gene Expression Data Society, specifies information to permit experimental reproducibility, standardization of data reporting and sharing, and allows global access to the original microarray data [9]. Annotation of the differentially expressed genes and classification into functional groups was done using the Database for Annotation, Visualization, and Integrated Discovery (DAVID) program (NIH).

### Northern blot confirmation of gene expression

Aliquots (10 μg) of total RNA were size fractionated on 1% agarose/formaldehyde gels as described previously [4]. After electrophoresis, gels were equilibrated in 1 M ammonium acetate and RNA was transferred to nylon membranes. Blots were baked for 2 hr at 80 C. Hybridization with cDNA probes was carried out at 42 C in ULTRAhyb hybridization solution (Ambion, Austin, TX) sequentially to random primed, ^32^P-labeled cDNAs of interest. Probes for TA1/LAT1, 4F2/CD98 and GAPDH were described previously [5]. The following partial cDNAs were generated by PCR from known positive template RNA to confirm expression patterns of additional genes of interest: rat GADD45, a 706 bp PCR fragment representing nucleotides 1 to 706 [GenBank NM_024127.1]; rat Insig1/CL6, a 1392 bp fragment representing nucleotides 339 to 1730 [GenBank L13619]; rat LAT2, a 990 bp fragment corresponding to nucleotides 580 to 1570 [GenBank NM_053442.1]. These partial cDNAs were PCR amplified under individually optimized conditions with template RNA from positive control rat tissues or cell lines and subcloned into pCR4 Blunt-TOPO plasmid vector. Rat p21 cDNA, a 316 bp fragment representing nucleotides 318 to 634 [GenBank U24174] was the kind gift of Dr. Phillip Gruppuso, RI Hospital. The identity of all cDNA probes was confirmed by DNA sequencing (Keck Biotechnology Laboratory, Yale University (New Haven, CT). Purified inserts were digested from vector with EcoR1. For LAT2, EcoR1 digestion resulted in two insert products and the larger, 660 bp fragment was used as probe. After hybridization and washing, blots were exposed to X-ray film in the presence of intensifying screens or a Cyclone Phosphorimager (Model A431201) (Downers Grove, IL).

### RT-PCR confirmation of gene expression

Semi-quantitative Reverse Transcription Polymerase Chain Reaction was used to assess relative expression of the following genes by amplification of these products: rat LDLr, a 531 bp fragment representing nucleotides 106 to 636 [GenBank NM_175762]; rat farnesyl diphosphate synthase (FDPS), a 656 bp fragment representing nucleotides 229 to 884 [GenBank NM_031840.1]; rat GADD45, a 706 bp fragment representing nucleotides 1 to 706 [GenBank NM_024127.1]; Insig-1, a 447 bp fragment representing nucleotides 1284 to 1730 [GenBank L13619] and rat GAPDH, a 452 bp fragment representing nucleotides 1369 to 1820 [GenBank NM_017008]. RNAs used for Northern blot analysis were used to generate cDNA and PCR products. Rat adult liver total RNA served as a positive control. Three micrograms of total RNA in a final volume of 20 μl was DNase I (Invitrogen, Carlsbad, CA) treated at room temperature for 15 minutes and the reaction stopped by addition of 25 mM EDTA followed by incubation at 65 C for 10 minutes to inactivate the DNase I enzyme. DNase treated total RNA was reverse-transcribed with and without reverse transcriptase for 60 minutes at 50 C using Superscript III Reverse Transcriptase (Invitrogen, Carlsbad, CA), Oligo (dT)_12–18 _(Invitrogen, Carlsbad, CA), RNase OUT ribonuclease inhibitor (Invitrogen, Carlsbad, CA, and 10 mM DNTP set (Invitrogen, Carlsbad, CA. Platinum Taq DNA polymerase (Invitrogen, Carlsbad, CA) was used for the RT-PCR reactions, which included 100 ng cDNA per 20 μl containing a final concentration of 1.5–2.25 mM MgCl_2 _0.2–0.3 mM dNTP, 0.5 μM each forward and reverse primers, and 0.025 U Platinum Taq DNA polymerase. PCR reactions were run in a Px2 Thermal Cycler (Themo Electron Corp.). LDLr and FDPS reactions were 94 C for 60 s followed by 94 C for 30 s, 55 C for 30 s, 72 C for 30 s (30 cycles), and a final incubation at 72 C for 10 minutes. GADD45 reactions were cycled at 94 C for 60 s, 50 C for 45 s, 72 C for 90 s for (40 cycles) and a final incubation at 72 C for 10 minutes. Insig-1 reactions were cycled at 94 C for 30 s, 55 C for 30 s, 72 C for 90 s (35 cycles) and a final incubation at 72 C for 10 minutes. GAPDH was cycled at 94 C for 60 s, followed by 94 C for 30 s, 53 C for 30 s, 72 C for 30 s (30 cycles), and a final incubation at 72 C for 10 minutes. Electrophoresis of PCR products was performed in 2% agarose, 1× TAE gel and visualized by ethidium bromide staining. Densitometry was run on LabWorks Software and values were ratioed to GAPDH densitometry values.

## Abbreviations

ER, endoplasmic reticulum; LAT1, L-type amino acid transporter, type 1

## Declaration of competing interests

The author(s) declare that they have no competing interests.

## Authors' contributions

HWL carried out portions of the bioinformatic analysis, submitted the MIAME dataset to GEO, prepared manuscript figures and tables for the original manuscript and participated in the initial text draft. CS prepared the cRNAs and conducted the microarray hybridization, scanning, raw data collection and supervised primary bioinformatic data analysis. ALB isolated total RNA from cell cultures, conducted Northern blot hybridization, generated PCR probes, RT-PCR quantitation and figure preparation, and participated in data analysis. BL participated in the initial microarray bioinformatic analysis, RT-PCR and data interpretation. CF carried out the cell culture and participated in figure preparation and unpublished confirmatory experiments. ES conducted additional bioinformatics analysis assessing specific classes of genes including stress response, cholesterol biosynthesis and other categories for a table in the revised manuscript; NLT designed experiments, supervised the overall project and prepared the final version of the manuscript for submission.

## Supplementary Material

Additional file 1 Major functional classes of arginine-sensitive genes in normal hepatic cells. GeneSpring functional categories of arginine-responsive genes in normal hepatic cells based on 9-way comparison of three independent data sets. Numbers of genes in each class and subclass are shown in parentheses along with gene accession numbers. Increase (I) and Decrease (D) defined as a two fold or higher change in expression from + arg to - arg. Click here for file

Additional file 2 Major functional classes of arginine-sensitive genes in hepatic tumor cells. GeneSpring functional categories of arginine-responsive genes in tumorigenic hepatic cells based on 9-way comparison of three independent data sets. Numbers of genes in each class and subclass are shown in parentheses along with gene accession numbers. Increase (I) and Decrease (D) defined as a two fold or higher change in expression from + arg to - arg. Click here for file
